# Editorial: From childbearing to childrearing: Parental mental health and infant development

**DOI:** 10.3389/fpsyg.2022.1123241

**Published:** 2023-01-05

**Authors:** Sandra Nakic Radoš, Susan Ayers, Antje Horsch

**Affiliations:** ^1^Department of Psychology, Catholic University of Croatia, Zagreb, Croatia; ^2^Centre for Maternal and Child Health Research, School of Health and Psychological Sciences, City, University of London, London, United Kingdom; ^3^Institute of Higher Education and Research in Healthcare-IUFRS, Faculty of Biology and Medicine, University of Lausanne, Lausanne, Switzerland; ^4^Department Woman-Mother-Child, Faculty of Biology and Medicine, Lausanne University Hospital, Lausanne, Switzerland

**Keywords:** perinatal period, mental health, child development, mothers, fathers, infant, parenting

Every day, around 385,000 babies are born worldwide. Childbirth is culturally perceived as positive, yet it may be a challenging experience for mothers and fathers. It is estimated that up to one-third of parents have psychological difficulties during pregnancy and postpartum. These difficulties then often affect the relationships between the mother, co-parent, and infant. As the relationships and interactions with both parents are crucial for infant development, parental mental health difficulties may have adverse effects on the family dynamics and the infant. Furthermore, infant characteristics can also affect the relationships and interactions with their parents, making these interactions complex and important to investigate.

This Research Topic “From Childbearing to Childrearing: Parental Mental Health and Infant Development” presents 15 papers - 14 original quantitative studies and one narrative review - examining the associations between parental mental health and different parenting and infant outcomes. Of the original studies, two used experimental designs, six studies had longitudinal and six cross-sectional designs. All studies included mothers, and one study included both mothers and fathers. Regarding the geographical distribution of the papers, 10 papers were from Europe, one from North America and four were international collaborations between researchers from Europe, North America, and Asia. These are summarized below.

## Maternal mental health in relation to infant behavior

Maternal mental health is intertwined with infant behavior problems. It was shown that mothers who report infant behavior problems also report more depression and anxiety symptoms and more mother-infant bonding problems in the first 6 months (Frankel et al.; Power et al.). However, this was established in cross-sectional studies, so the causality cannot be confirmed. Nevertheless, in a longitudinal study with mothers of preterm infants, maternal perception of the low infant self-regulation at 3 months predicted maternal depression symptoms at 6 months (Kmita et al.). Therefore, a bi-directional association between maternal mental health issues and infant behavioral or temperamental problems is probable.

Furthermore, it was reported that postpartum depression was related to the infant feeding method. However, no specific type of infant feeding method was a risk factor for postpartum depression *per se*. Other maternal experiences and infant-feeding cues played an important role for breast- and formula-feeding mothers (Kossakowska and Bielawska-Batorowicz). Mental health was intertwined with sleep, where poor sleep quality was both an antecedent and a consequence of impaired mental health. Mothers can be especially at risk because Sánchez-García et al. showed that mothers with children younger than 2 years had more disrupted sleep compared to the control group (women with children older than 6 years or no children). Mothers of infants were more likely to wake up more often during the night, report lower sleep quality, and sleep fewer hours, although different aspects of maternal sleep improved with infant's age.

## Parent-infant bonding as an aspect of parenting

Several papers looked into different predictive mechanisms for parent-infant bonding. First, Kalfon Hakhmigari et al. found a possible intergenerational mechanism where maternal recollection of her own parents' parenting was associated with maternal insecure anxious adult attachment style, which was in turn associated with poorer mother-infant bonding two months after childbirth. Other studies focused on parental mental health as a predictor of parent-infant bonding. It was found that fear of childbirth during pregnancy was predictive of a negative birth experience assessed two months postpartum, which was, in turn, predictive of poorer mother-infant bonding at 14 months (Seefeld et al.). The next two studies were consistent with this, showing that both postpartum depression and anxiety were associated with worse bonding. In this association, parental responsiveness had a mediating role in mothers and fathers (Nakić Radoš), while self-criticism was especially detrimental to mother-infant bonding in women with a history of depression or anxiety (Beato et al.). Therefore, it seems that some personality traits have a modifying role in these multi-layered associations.

## Parental mental health and infant outcomes

Maternal adverse experiences during her own childhood predicted more toddler emotional problems through their effect on maternal posttraumatic stress disorder (PTSD) symptoms (Ribaudo et al.). Also, maternal PTSD before or during pregnancy was associated with impaired peripartum mental health. Moreover, if mental health problems were comorbid with postpartum depression, mothers reported more feeding and sleeping problems in infants (Martini et al.). Maternal trait anxiety during pregnancy was associated with some infant development outcomes at 12 months (Jeličić et al.). An experimental study showed that exposure to acute maternal stress, measured with the experimental Caretaker Acute Stress Paradigm (CASP), affected infant autonomic nervous system regulation and behavior (Mueller et al.). Another experimental study with a longitudinal design showed that micro-temporal dyadic interaction patterns during the Still-Face paradigm in mid-infancy and maternal anxiety diagnosis predicted the development of insecure attachment in children aged 12–24 months. Moreover, an insecure attachment was associated with hormonal regulation in children at preschool age, showing a higher cortisol level during the stress paradigm compared to the children with secure attachment (Müller et al.).

## Interventions for parents and children

The final two papers dealt with early interventions for parents with infants and toddlers. Infant mental health treatment provided at home, on a weekly basis, to parents who reported depression symptoms, parenting stress, or a child's behavioral problems resulted in more positive socioemotional wellbeing of the child (Ribaudo et al.). Stolper et al. provided a narrative review of reviews and meta-analyses of interventions for parents with psychopathology aiming at disrupting the intergenerational transmission of psychopathology. The review first categorized risk and protective factors during the peripartum period into parental, family, child, and environmental domains. The review concluded that no universal intervention for prevention would work for all different families and settings. Instead, effective interventions should be individually tailored, focused on resources, addressing changeable risk factors by using different ways of delivery (individual, dyadic or group).

## Gaps in the knowledge and directions for future research

**Figure 1:**
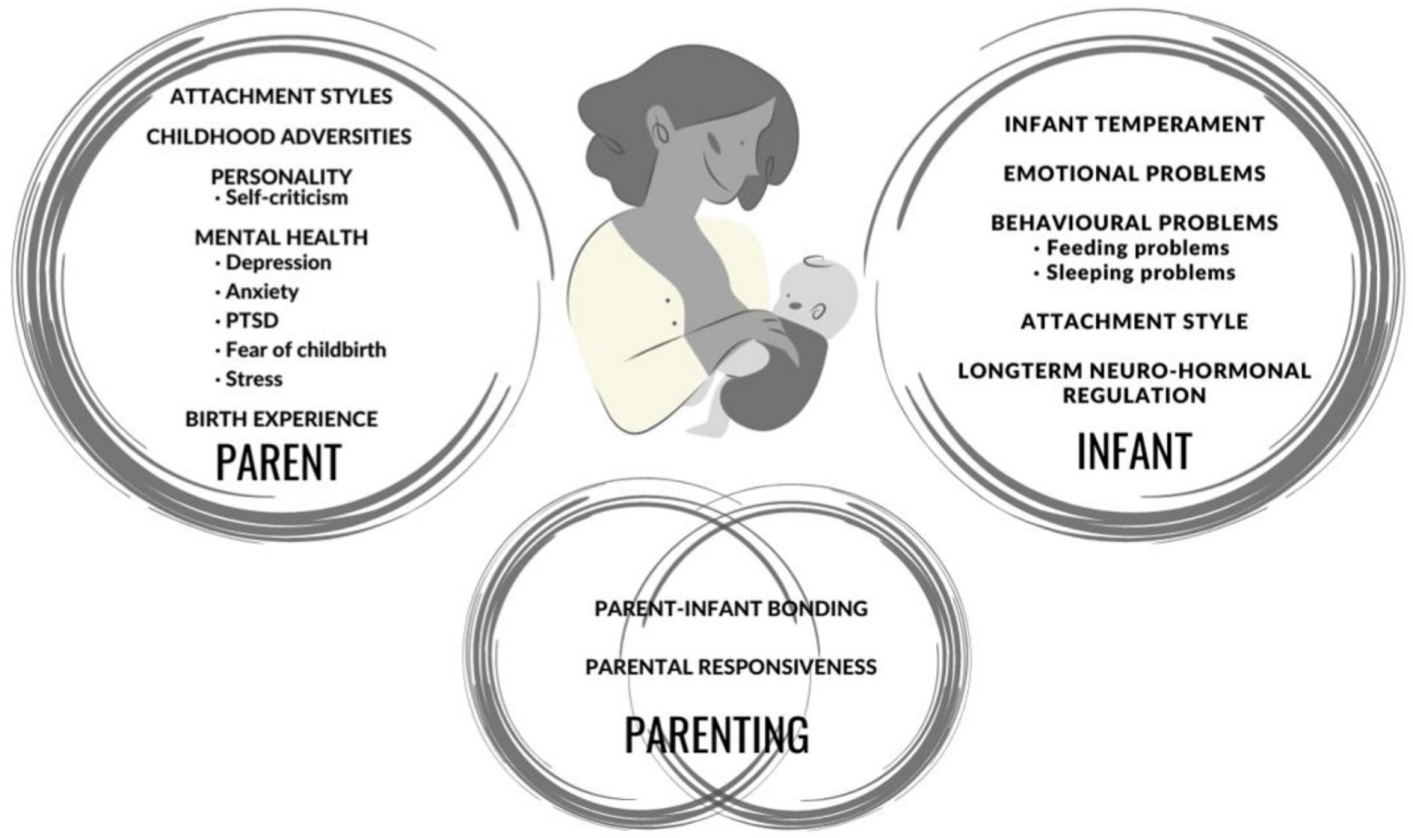
Schematic overview of the constructs examined within the Research Topic. (Figure created in Canva).

The papers in this Research Topic and overview of the different variables measured in these studies in Figure 1 illustrate the need for greater consideration and understanding of the complexity of relationships between maternal, infant and parent-infant factors. These include pre-birth factors, such as maternal childhood adversity and health; epi-genetic factors, such as the intergenerational transmission of trauma; birth factors, such as complications and trauma; early environment; and ongoing parent-infant and parent-child interactions.

Theories are important to underpin and guide this research, particularly when trying to understand complex relationships such as those between parental mental health and infant outcomes, as well as inter-dependent outcomes, such as attachment style and infant emotional or behavioral problems. Relevant theories that have been applied to this area include the biopsychosocial approach (Blount et al., 2021), which encourages consideration of biological, psychological and social factors in maternal and infant outcomes. The importance of taking a biopsychosocial approach is evidenced by research showing the brain basis of early parent-infant interactions (Swain et al., 2007); intergenerational transmission of trauma (Bowers and Yehuda, 2016), and influence of stress during pregnancy on neonatal behavior (Rieger et al., 2004). An updated dynamic biopsychosocial model needs to be extended to consider the dynamic nature of systems that influence our health. The updated dynamic model proposes that health outcomes are due to reciprocal influences of biological, psychological, interpersonal and macrosystem contextual dynamics (Lehman et al., 2017). It also considers how these influences may vary for different individuals over time. This seems particularly relevant to understanding the complexity of parent and infant interactions and outcomes over the course of infant and child development.

Methodologically, a biopsychosocial or multi-system approach requires multi-method, whole family, longitudinal studies that recognize the importance of the father/partner and couple's relationship (whether co-habiting or separate) in infant outcomes and development (Bergunde et al., 2022). It would also be useful to widen this to include co-habiting family members such as step-parents or grandparents, which was highlighted by a review of risk factors for child maltreatment (Ayers et al., 2019). More large longitudinal cohort studies are therefore needed in this area, such as the Dresden study on parenting, work, and mental health (Kress et al., 2019) and planned UK Early Life Cohort Study (Early Life Cohort, 2020), which provide multi-method, whole-family, longitudinal birth cohort studies. Such studies have the potential to generate a wealth of knowledge and understanding of biopsychosocial factors associated with infant outcomes and child development.

Also, common with research in other areas, the majority of research in perinatal mental health and infant outcomes is from high-income Western countries where samples are skewed toward White women, well educated, and higher income families, even when the population is more diverse. Thus, it is important to address gaps in knowledge in relation to minority groups and diversity.

In conclusion, this Research Topic presents a range of papers covering different aspects of the relationship between parental mental health and infant development, as shown in Figure 1. It highlights the complex dynamic systems and context, which are likely to influence infant development, and has identified ways in which future research can examine this to increase our knowledge and understanding.

## Author contributions

SNR initiated the Research Topic. SNR, SA, and AH were topic editors and wrote the manuscript. All authors contributed to the manuscript revision, read, and approved the submitted version.
